# Changes in hepatitis A virus (HAV) seroprevalence in medical students in Bangkok, Thailand, from 1981 to 2016

**DOI:** 10.1186/s13104-018-3733-7

**Published:** 2018-09-03

**Authors:** Palittiya Sintusek, Pattaratida Sa-nguanmoo, Nawarat Posuwan, Vorapol Jaroonvanichkul, Arnont Vorayingyong, Yong Poovorawan

**Affiliations:** 1 Division of Gastroenterology and Hepatology, Department of Pediatrics, Faculty of Medicine, King Chulalongkorn Memorial Hospital, Chulalongkorn University, Bangkok, Thailand; 2 Center of Excellence in Clinical Virology, Faculty of Medicine, Chulalongkorn University, Bangkok, Thailand; 3 Medical Education Unit, Faculty of Medicine, Chulalongkorn University, Bangkok, Thailand; 4 Department of Preventive and Social Medicine, Faculty of Medicine, Chulalongkorn University, Bangkok, Thailand; 5 Pediatric Liver Diseases and Immunology STAR (Special Task Force for Activating Research), Department of Pediatrics, Faculty of Medicine, Chulalongkorn University, Bangkok, Thailand

**Keywords:** Hepatitis A, Vaccine, Medical student, Vaccination record, Epidemiology

## Abstract

**Objective:**

This study aimed to determine the seroprevalence of anti-HAV IgG in Thai medical students in 2016 compared with the previous data and to demonstrate the cross-effective strategy to screen HAV seropositivity.

**Results:**

Sera from 176 first-year medical students (age 19.07 ± 0.59 years; 50% female) at a university hospital in Thailand were tested for anti-HAV IgG. Data from HAV vaccination records and questionnaires were also collected. HAV seropositivity was unexpectedly high (62.5%, n = 110). 37.5% (n = 66) had an HAV vaccination record. Of these, 60.6% received the full HAV vaccination series, 4.5% received one HAV vaccination, 34.8% did not receive HAV vaccination, and 3.0% had natural HAV immunity. The long-term efficacy of HAV vaccination was at least 97.5% over a mean of 15.55 ± 2.44 years. There was a significant difference in immunity between students with (66.7%) and without (50.9%) vaccination records (P = 0.028). Most of the student’s parents had a bachelor’s degree or higher (87.9%; n = 272) and above average income (mean 17,000.76 ± 194.22 USD/person/year). Parental education and socioeconomic status influenced vaccination accessibility in these medical students. Screening of vaccination records instead of routine anti-HAV IgG testing is a cost-effective and reliable strategy to determine HAV immunity in medical students in Thailand.

## Introduction

Hepatitis A virus (HAV) infection is the major cause of acute viral hepatitis worldwide, including Thailand [1]. Unlike hepatitis B and C virus infection, patients with HAV infection can easily be infected via ingestion of contaminated food and water. The clinical manifestations are not chronic but can cause debilitating symptoms and acute liver failure, especially in immunocompromised patients with chronic liver diseases and the elderly [2]. As a result, it is important to be aware that nosocomial HAV infection can be spread by person-to-person contact in hospitals, from healthcare workers to patients or vice versa [3].

The prevalence of HAV infection in developing countries is high due to inadequate sanitation and poor personal hygiene. In the past, HAV was endemic in Thailand, but the rate of HAV infection has been declining, especially in the young, which has led to a large seronegative population [4]. Thus, there is a growing population of susceptible adolescents and adults who tend to be symptomatic when outbreaks occur.

Health care personnel working in hospitals are considered as a high-risk group. Active immunoprophylaxis is recommended to prevent HAV outbreaks in hospitals. However, universal vaccination for all is costly and unnecessary for seropositive persons. Anti-HAV IgG testing has been suggested, and subsequent immunization has been encouraged for all seronegative individuals [5, 6].

Our study aimed to determine the seroprevalence of anti-HAV IgG in medical students and compare the results with those collected in 1982, 1992, 1996 and 2001 [1, 7, 8]. The trend of anti-HAV seropositivity can be used to develop a cost-effective strategy to determine HAV immunity among students (rather than using a mass screening program involving anti-HAV IgG testing) for helping to prevent an outbreak at the university.

## Main text

### Methods

Sera samples from first-year medical students at Chulalongkorn University collected between March, and June 2016 were screened for the protective antibody against HAV infection. All available medical student vaccination records were assessed for the timing and number of HAV vaccines that the students received. Medical students and their parents received questionnaires regarding each student’s history of HAV vaccination or natural infection and demographic data (including place of birth, parental education, and parental income).

Results of this HAV seropositive prevalence were compared with the results of our three previous studies which were conducted in 1982 [1], 1992 [7], 1996 [8] and 2001 [8] to better understand the current burden of HAV. In 1982, Viranuvatti et al. [1] reported the status immunity in Thai population (N = 1083) and included second-year (N = 153) and third-year medical students (N = 162) in the study. In 1992, Poovorawan et al. [7] also reported HAV seropositivity in 5 population groups that included fifth-year medical students (N = 35) in Bangkok. In 2001, Chatchatee et al. [8] determined HAV seropositivity of fourth-year (N = 60) and fifth-year medical students (N = 75) in 1997 and 2001, respectively and compared the data with previous studies by Viranuvatti et al. [1] and Poovorawan et al. [8]. These previous studies excluded participants who had received HAV vaccine. As a result, the studied demonstrated only the HAV seropositivity from natural infection. To detect HAV seropositivity, all previous used ELISA kits for anti-HAV IgG (Abbott Laboratories, North Chicago, Ill) that had sensitivity and specificity to detect anti-HAV IgG of 100% and 99%, respectively were used.

### Ethical considerations

First-year medical students with blood tests as a compulsory routine check-up for hepatitis B markers (anti-HBs, anti-HBc and HBsAg) and anti-varicella zoster virus (VZV) IgG provided written consent to use the rest of the sera sample for anti-HAV IgG testing. Ethics approval was granted by the Ethics Committee, Faculty of Medicine, Chulalongkorn University (IRB Number: 614/60).

### Laboratory methods

Anti-HAV IgG was assessed by enzyme-linked immunosorbent assay (DIA.PRO, Diagnostic Bioprobes Srl, Milan, Italy) following the manufacturer’s instructions. The sensitivity and specificity of the tests were 100% and 100%, respectively. These results were reported as seropositive or seronegative.

### Statistical analysis

All statistical analysis was performed using SPSS version 24.0.0 (SPSS, Inc, Chicago, IL, USA). Continuous and categorical data were presented as mean ± SD. Unpaired t-tests and Chi squared tests were used for between-group comparisons of the continuous and categorical data, respectively. A *P*-value < 0.05 was considered statistically significant.

### Results

#### Demographic characteristics

There were 176 medical students recruited into the study (mean age: 19.07 ± 0.59 years; 50% female) and 126 (71.6%) students completed and returned the questionnaires. These questionnaires demonstrated that most of the students’ parents graduated with a Bachelor’s degree or higher (n = 272, 87.9%) and the mean annual income per capita was 17,000.76 ± 194.22 USD (the mean annual income per capita in Thailand is 5901.40 USD [9]). Student’s demographic data and their parents’ careers and education levels are shown in Table 1.

**Table 1:** Students' demographic characteristics and parents’ careers and education levels

Characteristics	Mean ± SD or N (%)
Data from all students (n = 176)	
Age (years)	19.07 ± 0.59
Gender, female	88 (50)
Domicile	
Central	115 (65.3)
North	11 (6.3)
East	31 (17.6)
West	4 (2.3)
South	15 (8.5)
Data from HAV vaccination records (n = 66 records available)	
Received two injections (complete series)	40 (60.6)
Received one injection	3 (4.5)
Did not receive injections	23 (34.8)
Data from questionnaires (n = 126 questionnaires returned)	
Father’s career	
Businessman	53 (42.1)
Employee	31 (24.6)
Officer	18 (14.3)
Physician	9 (7.1)
Dentist	6 (4.8)
Pharmacist	3 (2.4)
Architect	2 (1.6)
Engineer	5 (4.0)
Pilot	1 (0.8)
Unknown	1 (0.8)
Mother’s career	
Businesswoman	42 (33.4)
Employee	22 (17.4)
Officer	30 (23.8)
Physician	4 (3.2)
Dentist	8 (6.3)
Pharmacist	2 (1.6)
Architect	1 (0.8)
Engineer	2 (1.6)
Housewife	11 (8.7)
Unknown	1 (0.8)
Salary (USD/month)	
Father’s (n = 101)	3345.9
Mother’s (n = 81)	2395.5
Father’s education	
Grade 1–6	4 (3.2)
Grade 7–12	5 (3.9)
Vocation certificate	1 (0.7)
Vocation diploma	1 (0.7)
Bachelor’s degree	80 (63.5)
Master’s degree	36 (25.6)
Ph.D. degree	4 (3.2)
Unknown	5 (3.9)
Mother’s education	
Grade 1–6	3 (2.5)
Grade 7–12	5 (3.9)
Vocation certificate	1 (0.7)
Vocation diploma	2 (1.5)
Bachelor’s degree	82 (65.1)
Master’s degree	34 (26.9)
Ph.D. degree	3 (2.5)
Unknown	6 (5.1)

#### Anti-HAV seropositivity and seronegativity

Among the medical students, 110 (62.5%) had anti-HAV seropositivity. Sixty-six (37.5%) students had their HAV vaccine history documented in vaccination records. Of these, 40 (60.6%) received two HAV vaccine injections (complete HAV vaccine series), 3 (4.5%) received one HAV vaccine injection, and 23 (34.8%) did not receive any HAV vaccine injections. Of the 23 that did not receive any HAV vaccines, 2 (8.7%; 3.0% of those who had vaccination records) had natural HAV immunity.

The long-term seroprevalence (at or above a protective level) among those who received the completed HAV vaccine series was 97.5%. The mean duration between the first HAV injection and anti-HAV IgG testing was 15.55 ± 2.44 years. For students whose vaccination records were not available, 56 (50.9%) had HAV immunity. There was a significant difference in detectable HAV immunity between medical students who had vaccination records and those who did not (Table 2).

**Table 2:** Hepatitis A virus (HAV) immunity among students

Characteristics	Vaccination records		P-value
	Available (n = 66)	Unavailable (n = 110)	
Overall HAV immunity	66.7% (44/66)	50.9% (56/110)	0.028
Overall HAV nonimmunity	33.3% (22/66)	49.1% (54/110)	
HAV immunity among those who received two HAV injections	97.5% (39/40)	–	
HAV immunity among those who received one HAV injection	100% (3/3)		
Natural HAV immunity among the unvaccinated	8.7% (2/23)		
Time from the first HAV injection to anti-HAV IgG testing	15.55 ± 2.44 years		

#### Changing epidemiology of HAV seropositivity among medical students over time

**Fig. 1:**
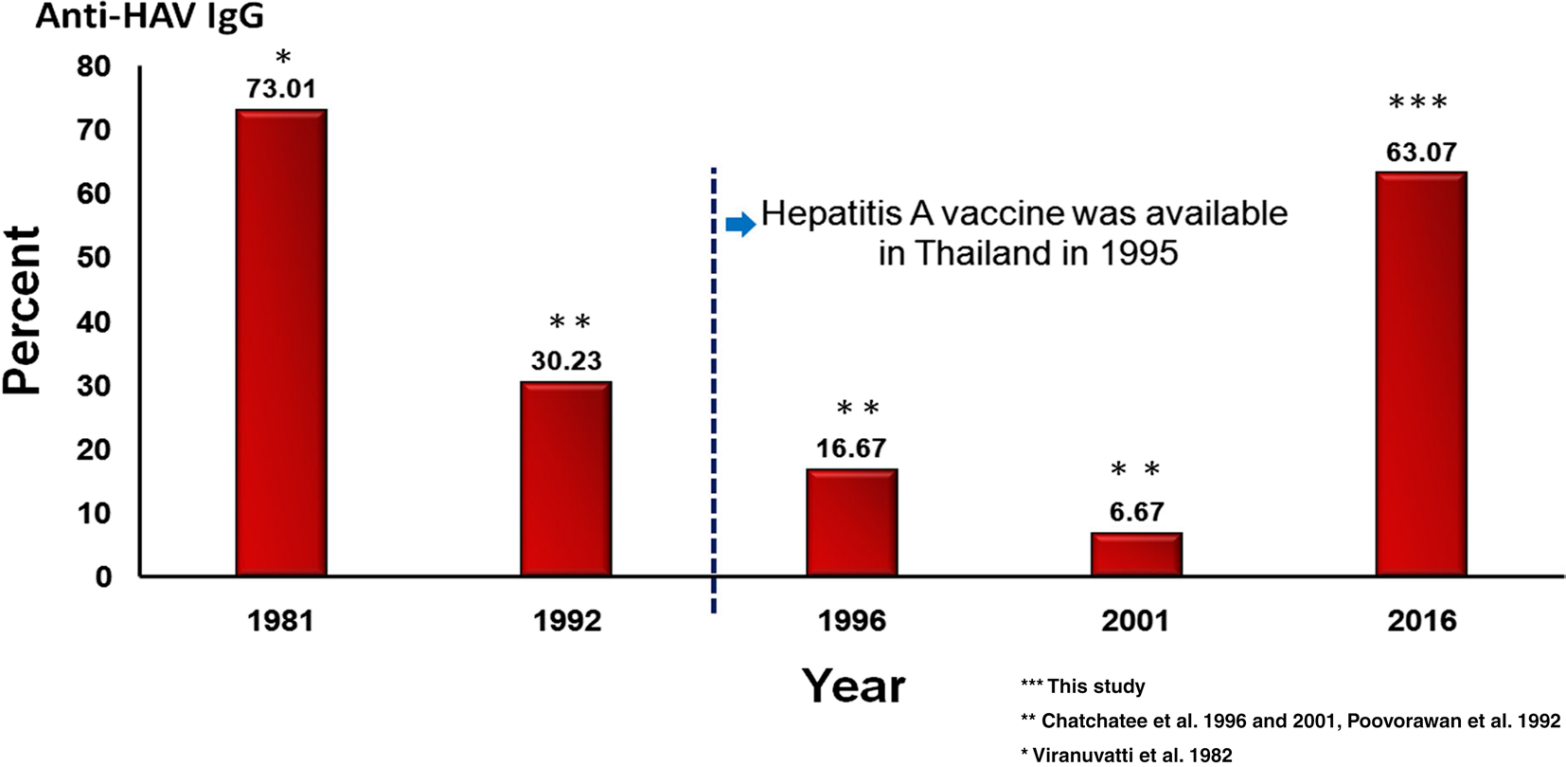
Seroprevalence of HAV immunity among Thai medical students

As shown in Fig. 1, according to the comparison involving the results of our previous studies in 1982 [1], 1992 [7], 1996 [8] and 2001 [8], the prevalence of HAV seropositivity dramatically increased in 2016, approximately two decades after the HAV vaccine was made available worldwide (in 1995). According to the group of medical students who had vaccination records (N = 66), the high HAV seropositivity in 2016 was due to HAV immunization. This contrasts with earlier changes in anti-HAV seroprevalence, which reflected natural HAV immunity. The dynamic changes show that seropositivity of 73.01% in 1982 [1] decreased to 30.23% in 1992 [7], 16.67% in 1996 [8], 6.67% in 2001 [8] and to 8.7% in the present study.

### Discussion

The prevalence of HAV seropositivity from natural infection in medical students has seen a significant decrease over time from 73.01% in 1982 [1] to 6.67% and 8.7% in 2001 [8] and 2016, respectively. This dynamic change confirms that over time, HAV enmity in Thailand has shifted from low to very low due to the improvement of sanitation and national economy [10]. The present study demonstrated the overall high HAV seropositivity in medical students in this era resembled that of the data in 1982 [1] when the vaccine was not available. HAV immunization was the only explanation for the high HAV seropositivity in the present study. According to a report from the Thai Ministry of Public Health (MoPH) [11, 12], the HAV infection rate each year has not increased except when sporadic outbreaks have occurred which increased the infection rate above the background rate, and there has been no record of an HAV outbreak in medical students that caused high HAV seropositivity referring to the present study. As the Thai MoPH officially launched its nation-wide Expanded Program on Immunization (EPI) in 1977 which was designed to cover the entire population in all parts of Thailand, all government hospitals and health care centers must provide all immunization in the EPI schedule to all people (both children and adult) for free of charge. To improve the immunization services, the Advisory Committee on Immunization in Thailand (consisting of 28 experts in immunization and related fields) who developed written recommendations to Thai MoPH regarding vaccines and implement EPI into the Thai basic health services continuously studies the researches related to vaccines, epidemiology of vaccine-preventable diseases and the rate vaccine coverage. Unfortunately, unlike the vaccines in Thai EPI including tuberculosis, hepatitis B, diptheria-pertussis-tetenus, poliomyelitis, measles and Japanese encephalitis vaccine, there is no record of the number of optional HAV vaccinations administered nationally between 1995 and 2016 that could strongly support the high HAV seropositivity in these medical students. The only evidence of high HAV seropositivity in medical students that could be from immunization was the student subgroup data of those who had vaccination records in our study (N = 66) in which 65.1% of seropositivity received HAV vaccine while only 8.7% of seropositivity from natural infection. The high parental education and socioeconomic status might explain optional vaccine accessibility in these medical students.

However, there was also a high incidence of HAV seronegativity in 43.1% (N = 176) of all and 49.1% (N = 66) in a subgroup of medical students who did not have vaccination records. As medical students have a risk of exposure to infectious agents including HAV, a strategy to detect the immunity is needed. HAV vaccine and other vaccine-preventable diseases should be managed by immunization when seronegative students start practicing in hospitals.

In our university hospital, for more than a decade, have implemented a policy of routinely checking immunity to hepatitis B and Varicella virus among the first-year medical students. Seronegative students then receive hepatitis B and Varicella vaccines. Serologic testing for anti-HAV IgG is not routinely carried out among medical students due to the high cost of HAV vaccines and no significant benefit for HAV vaccination with screening from cost–benefit analyses studied in Thailand [13], which makes them unattainable for seronegative students. However, regarding the parents’ socioeconomic status and education levels in the present study, the vast majority of the parents had high income and education when compared with general Thai population. Since medical students are one of the most high-risk groups, this informative data will aid and convince parents to consider and take action to get all proper vaccines for their children if these children are found to have seronegativity after routine checking by the university hospital. As a result, university hospitals across the country should routinely check all serology for vaccine-preventable diseases without hesitation to best inform medical students and their guardians to proceed with adequate vaccinations in order to prevent any diseases during a student’s course of studies. Furthermore, Screening of vaccination records was very useful and very reliable when we correlated them with serologic anti-HAV IgG results. Screening of vaccination records might be an easy and cost-effective way to initially evaluate vaccine-preventable immunity against HAV and other infections before serology testing. Pediatricians should advise parents to keep their children’s vaccination records as long as possible, as they will be very useful when their children attend school or enter the workforce in the future.

In conclusion, the present study, the seroprevalence of HAV immunity among Thai medical students in 2016 was very high compared to the previous seroprevalence trend. HAV immunization can explain this high seroprevalence, rather than natural HAV infection. Vaccination records were shown to be important, as documented vaccinations had a good correlation with the results of anti-HAV IgG testing. Parents’ high levels of education and income may explain the high level of access to HAV vaccines. Screening of vaccination records before anti-HAV IgG testing or HAV immunization can be considered a cost-effective method for preventing HAV infection among medical students in Thailand and other countries with a similar pattern of HAV epidemiology.

## Limitations of the study

No socioeconomic, parental education or vaccination data for medical students from the previous studies were available for comparison. The ELISA kit used to determine the anti-HAV IgG in the present study was different from the previous study. However, the diagnostic values following the manufacturer’s data were excellent in both ELISA kits with the sensitivity and specificity > 99%.

## Abbreviations

HAV: hepatitis A virus
ELISA: enzyme-linked immunosorbent assay
